# Revalidation of preservice physical education teacher’s teacher identity scale in Chinese physical education student teachers

**DOI:** 10.3389/fpsyg.2022.1061215

**Published:** 2023-01-10

**Authors:** Anlu Yang, Yongshun Wang, Lei Yao, Xiaofen D. Hamilton

**Affiliations:** ^1^School of Education, Beijing Sport University, Beijing, China; ^2^College of Physical Education, Huaqiao University, Quanzhou, China; ^3^Department of Curriculum and Instruction, The University of Texas at Austin, Austin, TX, United States

**Keywords:** teacher identity, teacher identity scale, identity measure, reliability and validity, physical education student teachers

## Abstract

**Introduction:**

Teacher identity (TI) is a crucial aspect to pre-service physical education teachers (PPETs) to becoming high quality teachers and plays an important role in their career choice. Thus, a deep understanding of their TI may help improve the quality of future PE teachers. This study aimed to provide further evidence of reliability and validity for Preservice Physical Education Teacher’s Teacher Identity Scale (PPET-TI scale).

**Methods:**

The study went through a three-stage development process: (a) examining the content validity of original scale; (b) data collection using a sample of student teachers in physical education; (c) re-test the reliability and validity of the scale developed by Zhang.

**Results and Discussion:**

After having re-examined the content validity, the domain of affects was deleted. The modified scale then consisted of the three domains (i.e., value and expectation, perceived confidence of teaching PE, and professional growth). A total of 944 physical education student teachers in China participated in the study. Confirmatory factor analysis (CFA) was employed to investigate the construct validity, the results suggested the data did not fit the original model. After the unfitted item was excluded. All CFA fit indices were within the acceptable range. Reliability of the scale was then examined by calculating G-C alphas. The alpha values were within the acceptable range. The modified model was reliable and valid. The revalidation of the scale provided more us with a scale that could produce reliable and valid score measuring PPETs’ TI.

## Introduction

The world’s growing population has brought the increasing in school-age population. As such, the need for new teachers will continue to increase (Heffernan and Newton, 2019). However, the enrollment rate of teacher education is declining (US Department of Education, 2019). Moreover, teacher education graduates are generally reluctant to devote themselves to teaching career in general (Berry and Shields, 2017). The same phenomenon has also been found in physical education (Woods and Ayers, 2019). Previous research has found that there is a shortage of physical education teachers in the US (Ward, 2019), China (Anna et al., 2021), and other countries (Melnychuk et al., 2011; Vámos and Doczi, 2015; Du, 2019). Alarmingly, the enrollment in PETE programs has declined in recent years (Keating et al., 2017). Therefore, it is particularly important to explore what factors influence preservice teachers’ career choice (Ferry, 2018; Liu and Keating, 2022).

In the past 20 years, researchers began to realize the importance of teachers’ teacher identity (TI) in teachers’ career choice and professional development (Hong, 2010) given that the number of studies on the topic is increasing (Barkhuizen, 2017; Keating et al., 2017; Wang and Zhang, 2021). As noted by Keating and colleagues in a systematic review (Keating et al., 2017), a few different definitions of TI have been used in the literature. However, it is widely believed that TI is a way of being and becoming a teacher and is a continuous process of integrating both “personal” and “professional” elements (Beijaard et al., 2004). Puchegger and Bruce (2021) have suggested that TI is a fluid, dynamic identity performance that interacts with the teaching context. In other words, the authors pointed out that different teaching context would affect the TI of physical education teachers.

Existing research on the topic has suggested that TI is strongly related to many factors that play an important role in teaching and learning, such as teachers’ beliefs (Wrench and Garrett, 2012), understanding educational changes (Vähäsantanen, 2015), self-efficacy (Wagoner, 2015; Azim, 2017), job burnout (Samuel and Stephens, 2000), and work engagement (Zhang et al., 2018). More importantly, many researchers indicated that TI is shaped in teacher education programs and is refined during their teaching career (Beijaard et al., 2004; Beauchamp and Thomas, 2009). A lack of attention to TI in educational research and teaching practice would be detrimental to teachers’ professional growth, teaching quality, and job satisfaction (Day and Gu, 2007).

### Previous research on TI of pre-service physical education teachers

As teacher candidates, the quality of pre-service teachers will directly influence the quality of education in general and teachers in particular. Their TI is particularly important in meeting the growing demand for new qualified teachers (Adams et al., 2006). The TI of preservice physical education teachers (PPETs) developed determines what their attitudes are, where they strive for, whether and how they seek out professional development opportunities, and which obligations they see as inherent to their role (Chong et al., 2011). Unfortunately, while increasing attention has been given to TI in general, much fewer studies have been conducted on PPETs’ TI (PPET-TI). Research on this topic is still rare (Liu, 2019; Liu and Keating, 2022).

Among the available studies on PPET-TI, most focused on how TI are formed and what the determinants of TI are (Keating et al., 2017). Through a review of the existing research, it was found that PPET-TI development is influenced by three major factors: (a) physical education teacher education (PETE) program or the professional environment (Dowling, 2011; Ferry, 2018; Puchegger and Bruce, 2021). Ferry (2018) found that the effects of PETE programs on PPET-TI were limited and PETE programs should take into account the impact of acculturation so that PPETs could be better prepared for their future careers. Puchegger and Bruce (2021) found that PETE programs should explicitly address and develop student teachers’ understandings that developing physical education TI is a fluid and ongoing process. (b) sport competence, body image or physicality (Sirna et al., 2010; Wrench and Garrett, 2012; Virta et al., 2019). Wrench and Garrett (2012) found that those who were successful in sports, or participated in organized sports in childhood had a positive and strong commitment to be a physical education teacher. Virta et al. (2019) show that the conceptions of body and physicality represent a central element of physical education teachers’ TI. And (c) student teaching (Solmon et al., 1991; Lee and Jo, 2016). As well documented in the literature, student teaching is the critical learning phase to completes the transition phase from PPETs to in-service teachers and PETE programs around the world seem to all have the component of student teaching (Rossi et al., 2016). Many studies have noted the importance of understanding the contribution of student teaching to PPETs’ TI formation (Solmon et al., 1991; Sirna et al., 2010; Margarida et al., 2012; da Cunha et al., 2014). Solmon et al. (1991) found that student teaching have a profound effect on PPET-TI construction, and student teachers who had vague self-images as a teacher prior tended to have clear self-images during student teaching. Margarida et al. (2012) analyzed one student PE teacher’s daily events and found that in the context of practicum, the relationship established with colleagues, students, and other teacher contributes to her understanding of being a teacher. da Cunha et al. (2014) noted that the university supervisors or physical educators play an essential role for forming PPETs’ TI during student teaching. In essence, student teachers are in a special professional development stage. Their level of TI will directly affect their career choice after graduation and teachers’ professional development after employment. As such, it is important to examine student teachers’ TI in PETE programs.

However, to date, our knowledge about essential elements and universal features of PPET-TI is very limited and the characteristics of PPET-TI have been overlooked (Keating et al., 2017). Among the previously reported studies on the topic, one study has investigated the characteristics of PPET-TI and answered two important questions “who am I” and “what do I do” (da Cunha et al., 2014). Another study summarized six characteristics (i.e., list them here) shared among PPETs through a review of the existing PPET-TI studies (Liu and Keating, 2022).

### Previous research on the development PPET-TI scales

The development of reliable and valid PPET-TI instrument is particularly essential in quantitative research because it enables researchers to conduct studies on PPET-TI with more confidence. A thorough search of the literature indicated that there were two studies on the instrument development of PPET-TI. Liu and Keating (2022) developed the PPET-TI scale based on the theoretical framework of dynamic systems model which is a metatheoretical framework about identity (Kaplan and Garner, 2017). The authors conceptualized TI as the extent to which someone describes themselves as a teacher, commits to the teaching profession’s expectations, and “considers being a teacher an important aspect of who they are” (Kaplan et al., 2015, p. 4). The model consisted of three domains: self-definitions (assuming a teacher’s role and projecting a future professional position in physical education), teaching goals (aligning teaching goals with national standards), and professional responsibilities (a combination of ontological and epistemological beliefs and perceived action possibilities; Liu and Keating, 2022). The instrument demonstrated acceptable validity and reliability (see Liu and Keating, 2022 for the detailed information concerning the values of the fit indices).

The second PPET-TI scale was reported in Chinese (Zhang, 2017), which was based on physical education teachers’ TI scale developed by Zhou et al. (2012). Song’s (Song and Wei, 2006) Chinese general teachers’ teacher identity scale which used the theory of professional self (Kelchtermans and Vandenberghe, 1994) and Meyer et al.’s (1993) occupational commitment theory were taken into consideration to develop the physical education teachers’ TI scale by Zhou et al. (2012). The scale has five domains including professional value, affects, perceived confidence of teaching PE, sustainment and professional growth. The scale showed acceptable reliability and validity. Based on the scale of Zhou et al. (2012) and Zhang (2017) combined the results of interviews with eight PPETs and experts to develop the scale of measuring PPET-TI, which was validated with a sample of 1,277 PPETs from 10 universities. The 21-item scale consisted of the following domains: (a) value and expectation, (b) perceived confidence of teaching PE, (c) affection of PE as a professional career, and (d) planned professional growth. PPET-TI has also shown the evidence of acceptable reliability and validity, providing a useful means to collect data regarding PPET-TI in China (Zhang, 2017). Refer to Table 1 for detailed information concerning the structure of the scale and items in each domain.

**Table 1:** The structure and items in the original PPET-TI scale with four domains.

Domain	Items
value and expectation (6 items)	A1. Physical education is an important part of school education.
	A2. K-12school students should receive physical education
	A3. I hope to achieve something in my teaching career.
	A4. PE teachers play an important role in the all-round development of students.
	A5. I hope to cultivate students’ lifelong sports awareness through my teaching.
	A6. I hope that students can experience the enjoyment of physical activities through my teaching.
Perceived confidence of teaching PE (5 items)	B1. No matter what the working environment is, I can complete teaching physical education efficiently.
	B2. I am capable of handling the pressure from other subject teachers in my future work
	B3. I am gradually having the quality to be an excellent physical education teacher.
	B4. I have the confidence to solve the problems encountered in PE classes.
	B5. I can formulate teaching strategies according to the actual situation of students
Affects (5 items)	C1. I am willing to attend professional development learning opportunities related to PE
	C2. I am able to master various teaching skills taught in PE teacher education program.
	C3. I feel very happy to help students who do not love sports to actively participate in physical education.
	C4. I like the PE teacher education program.
	C5. I can become a qualified physical education teacher.
Professional growth (5 items)	D1. I pay attention to the information about the recruitment of physical education teachers.
	D2. To become a physical education teacher makes me full of energy
	D3. I often collect knowledge about physical education.
	D4. I pay attention to the teaching methods used by teachers in PE teacher education program.
	D5. I would like to communicate with PE practitioners frequently.

Overall, both scales were designed to measure PPET-TI with different domains. The common domains included in the two scales included expectation, and professional goals of teaching PE. Both included the current self-description as a PE teacher, and the future intention and actual behaviors. They lend insights into future quantitative research on PPET-TI in Chinese and English. Although the two different psychometric scales showed co-existence of measuring PPET-TI, there are several differences. Firstly, the scale developed by Liu and Keating (2022) in the US was theory-driven based on the theoretical framework of dynamic systems model. The Chinese scale by Zhang (2017) was also theory-driven, but it not directly theory-driven, it developed based on the previous theory-driven research on the topic. Secondly, the scale developed by Liu and Keating (2022) used a 7-point Likert-type scale, but a 5-point Likert-type scale was used in the scale reported by Zhang (2017). The psychometric literature suggests that having more scale points is better (Preston and Colman, 2000), but a 7-point Likert-type scale was used to increase response rate and response quality along with reducing respondents’ “frustration level” (Revilla et al., 2014). Thirdly, the scale of Liu and Keating (2022) measured PPETs’ teaching goals as an important aspect of TI while Zhang’s (2017) scale measured PPETs’ perceived teaching ability as an important aspect of TI.

### The purpose, questions, and hypothesis of the study

The noted earlier, student teaching is the most critical phase of PPETs’ professional preparation. Their TI is of concern as it might influence PPETs’ career choice and the quality of their teaching as in-service teachers. Therefore, the purpose of the current study was to validate the scale developed by Zhang (2017) among student teachers in PETE in China. The question that this investigation intends to answer whether the scale developed by Zhang (2017) fit the population of Chinese student teachers in PETE. Based on the above literature review on the topic, it was hypothesized that the survey developed by Zhang (2017) might not be suitable for the population of student teachers in PETE. There was a need to modify the domains or/and items embedded in each domain to fit the intended population.

## The significance of the study

A reliable and valid instrument/scale for measuring student teachers’ TI in PETE is always the first step for conducting high quality research on the topic. It is hoped that the outcome of the study could help us better examine the quality of PPETs’ preparation. It is also important to note that the scale developed by Liu and Keating (2022) is more representative of American characteristics in the domain of teaching goals, aligning it with Shape America’s national teaching standards for initial PE teachers, which is different from those in other countries. As such, some of the items in the scale were not appropriate for some countries. For example, in the domain of professional responsibilities, there were the item of beginning or maintaining an active membership of professional organizations related to PE, and the item of contribute to PE-related professional organizations (e.g., organizing events, fundraising, and donation). In China, the main responsibility of PE teachers is to teach physical education content in schools, and they are not closely connected to professional organizations (Wang and Liu, 2017; Chen, 2020). The Chinese scale (Zhang, 2017) does not seem to have such strong an emphasis on the connection with professional organizations and may be used by researchers from countries and regions with similar PE teachers’ professional focuses to those in China. Therefore, it is also hoped that the results of the current study would provide us with another scale that is more general without specific characteristics tied to certain countries’ uniqueness, providing baseline data for international comparisons, which was impossible prior to the completion of the study.

## Materials and methods

Research involving human subjects does not need an IRB approval in China. However, the common practice for protecting participants was followed in the current study. No personal identification information was used at any time. Participants were informed that their participation was completely voluntary, and they were free to withdraw from participating in the study at any time.

The study went through a three-stage development process: (a) examining the content validity of original scale; (b) data collection using a sample of student teachers in physical education; (c) re-test the reliability and validity of the scale developed by Zhang (2017). The following section is organized by the order when each stage was completed. And Table 1 displays the structure and items in the original PPET-TI scale.

### Stage 1: Re-examining the content validity of the original scale

In order to make better use of the PPET-TI scale across nations, it is necessary to examine the content validity of the original scale reported by Zhang (2017). An online survey was completed by six experts, three of them were from China while the others were Chinese American scholars in the departments of kinesiology and/or physical education in the US. These experts’ research expertise is related to PETE, TI, measurement and assessment in physical education. All of them are professionally trained in both Chinese and American universities, and understand both languages very well.

The experts provided feedback on the suitability of each domain and items embedded in each domain. All experts agreed on three domains (i.e., value and expectation, perceived confidence of teaching PE, and professional growth). The experts also agreed on the items included in each of the above domains. However, in the domain of affects, it showed a low agreement among experts, because some items are not associated with the domain of affects or some of them overlapped with the other domains. As such, this domain was deleted due to the low agreement among experts (i.e., <80%; Meyers et al., 2017). As a result, there were 16-items in total, with six items in the domain of value and expectation, five items in the domain of perceived confidence of teaching PE, and five items in the domain of professional growth.

### Stage 2: Revalidation of PPET-TI among student teachers in PETE

The purpose of the stage was to revalidate the scale among student teachers in PETE, which has not previously been done. Except for the difference in participant selection, all other steps were identical to those used in the two studies reported in the literature on the topic (e.g., Zhang, 2017; Liu and Keating, 2022).

### Participants

The participants (*n* = 944) of the revalidation study were physical education student teachers who were enrolled in 21 PETE programs in China. Due to the standardized schooling system implemented in China, all participants had a similar age (i.e., 22–23 years old) with a very small variance, preventing from generate any meaningful statistic power. Therefore, information about participants’ age was not collected. In addition, unlike that in many western countries, PPETs can also conduct their student teaching in the higher education settings where physical education is required for the first two years. In China, PETE programs are offered in three different types of colleges and universities: (a) kinesiology/sport institutes and universities, which aim to prepare professionals for the fields of sports and physical educations; (b) normal colleges and universities, focusing on PE teacher preparation; and (c) comprehensive universities for all kinds of future professionals concerning sports, physical education, fitness, and recreation. All teaching track majors need to have student teaching for one year in nearby schools and/or universities. The participants were from all the five regions (i.e., Southern, Northern, West, Eastern, and Central regions) in China with 552 (58.5%) males and 392 (41.5%) females. Among the PE student teachers, 404 (42.8%) came from kinesiology/sport institutes and universities, 264 (30%) from comprehensive universities, and 276 (29.2%) from normal universities. Detailed participant information is presented in Table 2. The reason for indicating the three types of colleges and universities for PETE was the differences in the curriculum for PPETS.

**Table 2:** Demographic information of the participants.

Category	Subcategory	Participants (%)
Gender		
	Female	552 (58.5%)
	Male	392 (41.5%)
Teaching grade		
	Elementary	345 (36.5%)
	Middle school	289 (30.6%)
	High school	209 (22.1%)
	College	101 (10.6%)
Type of University		
	professional sport university	404 (42.8%)
	normal university	264 (30%)
	comprehensive university	276 (29.2%)
Length student teaching		
	Less than 1 month	28 (3%)
	1–2 months	568 (60.2%)
	3–4 months	316 (33.4%)
	More than 5 months	32 (3.4%)
Internship weekly class hours		
	1–4 class(es)	152 (16.1%)
	5–8 classes	416 (44.1%)
	9–12 classes	284 (30.1%)
	13 or more classes	92 (9.7%)

Age is not included because all the participants had a similar age (22–23 years old) due to the standardized education system implemented in China.

### Revised PPET-TI-Chinese scale

As noted earlier, the revised scale consisted of three domains with a total of 16 items. These items were randomly listed in the survey with the common demographic info such as age and gender at the end. A 5-point Likert-scale was used to measure the strength of TI. No negative items were used.

## Data collection

An online survey link was sent to all student teachers enrolled in PETE programs from five regions includes 21 colleges and universities in China. The survey provided the participants with the following information: (a) the purposes of the study, (b) instructions and directions for completing the questionnaire, (c) the confidentiality information. The students completed the questionnaire online anonymously. No personal identification information was collected, so that the participants could answer the survey without any pressure. In addition, this study used various techniques to control response bias, such as screening short response time and score outliers (Curran, 2016). It was also ensured that the completed surveys had a different IP address to avoid duplicates.

### Data analysis

Descriptive statistics were first performed using SPSS version 26.0 (SPSS Inc., Chicago, IL). Scores generated by the PPET-TI-Chinese scale need to be evaluated to determine their internal consistency by calculating Cronbach Alpha (Meyers et al., 2017). The same alpha cut-off value (i.e., >0.70) used in the study of Zhang (2017) in the development of the original scale was employed, which was also used in the study done by Liu and Keating (2022). For the PPET-TI-Chinese scale test score factorial validity, confirmatory factor analyses (CFA) were performed using AMOS version 24.0 (SPSS Inc. Chicago, IL). Because maximum likelihood estimation was utilized to estimate the model fit in CFA, further evaluation of data assumptions related to multivariate normality is required (Meyers et al., 2017). This study checked univariate normality for each item first. Skewness and kurtosis reflect the normality of data from the symmetry and steepness of probability distribution. Thus, when the univariate skewness and kurtosis values of descriptive statistics do not exceed 1 (see Table 3), the univariate normality of the data is considered acceptable (Meyers et al., 2017). Then, the multivariate normality was examined using AMOS; the multivariate kurtosis was-0.189 (critical ratio = −0.093), indicating multivariate normality. The original model was then tested using CFA.

**Table 3:** Results of univariate normality test.

Item no	Mean (SD)	Skewness	Kurtosis
A1	3.56 (0.96)	−0.72	−0.01
A2	3.56 (0.97)	0.01	−0.15
A3	3.58 (0.99)	−0.63	−0.24
A4	3.56 (0.98)	−0.62	−0.19
A5	3.56 (0.99)	−0.65	−0.31
A6	3.57 (1.00)	−0.57	−0.41
B1	3.81 (0.97)	0.11	−0.27
B2	3.86 (0.96)	−0.56	−0.45
B3	3.83 (0.93)	−0.61	−0.29
B4	3.84 (0.93)	−0.59	−0.35
B5	3.81(0.96)	−0.48	−0.39
D1	4.01 (0.90)	−0.50	−0.47
D2	2.92 (0.82)	−0.37	−0.57
D3	4.02 (0.90)	−0.37	−0.62
D4	4.00 (0.92)	−0.41	−0.54
D5	4.03 (0.91)	−0.28	−0.36

In general, five indices are chosen to assess model fit using CFA: (a) chi square ($\chi^{2}$); (b) comparative fit index (CFI); (c) Tucker-Lewis Index (TLI), which is also called the non-normed fit index; (d) GFI; and (e) RMSEA (Ho, 2006; Meyers et al., 2017). These five absolute and incremental fitting measures are applicable to evaluate the model fit. However, χ2 index has very sensitive feedback to sample size and sample violation, so it is an inappropriate index when the sample size is large (West et al., 1995; Byrne, 2009). CFI, GFI and TLI measurement models are considered as relatively improved measures of fit, whereas RMSEA measures how well the parameters of a model fit the covariance matrix (Browne and Cudeck, 1992; Ho, 2006; Meyers et al., 2017). In terms of the cut-off values for the above indices, it is acceptable if the *χ*2/df ≤ 3 (Klein, 2004), CFI, TLI and GFI values are more than 0.90 or RMSEA values are less than 0.08 (Meyers et al., 2017; Keith and Reynolds, 2018). In addition, structure coefficients were examined to assess the data fit. In general, the structure coefficient needs to be greater than 0.30 to be included in the factor (Meyers et al., 2017). Of more importance, many researchers in psychometrics and applied statistics suggest that it is necessary to evaluate the overall data through the overall method and professional knowledge (McDonald, 1999; Ho, 2006; Meyers et al., 2017).

## Results

As noted earlier, there are two stages in the current study. Stage one was designed to validate the content validity using experts and its results were used to revised the domains and items for stage two. As a result, the results reported below are only generated by stage two.

### Reliability and validity of the 16-item scale

#### Reliability

G-C alphas for the domains of value and expectation, perceived confidence of teaching PE, professional growth, and the overall scale were 0.88, 0.87, 0.75, and 0.88, respectively. These values of alphas were all within the acceptable rage.

#### Construct validity

**Figure 1:**
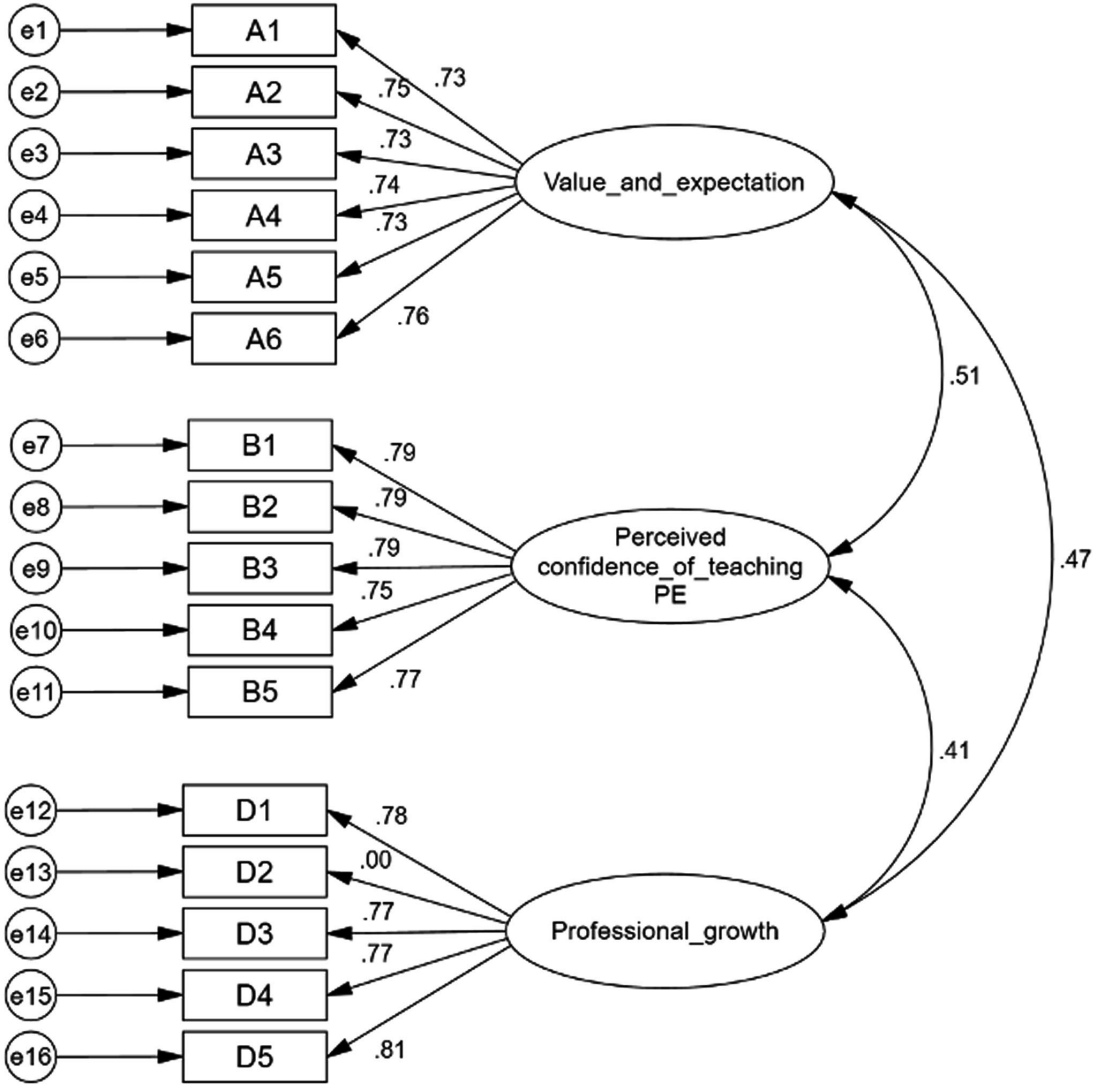
Original structure model of PPET-TI scale with four domains.

The CFA results testing the original structure are presented in Table 4. The measurement model is illustrated in Figure 1. All unstandardized factor loading were statistically significant and meaningful except D2 and all standardized factor loading were all greater than 0.30, except D2. Therefore, it was deemed necessary to delete D2.

**Table 4:** Factor loadings of original scale.

Factor	Item	Estimate	S.E.	C.R.	*p*	Standardize estimate
Value and expectation	A6	1.00				0.77
	A5	0.95	0.04	26.06	<0.001	0.73
	A4	0.93	0.04	25.15	<0.001	0.74
	A3	0.94	0.04	25.79	<0.001	0.73
	A2	0.95	0.04	24.14	<0.001	0.75
	A1	0.91	0.04	26.36	<0.001	0.73
Perceived confidence of teaching PE	B5	1.00				0.78
	B4	0.92	0.04	26.00	<0.001	0.75
	B3	0.97	0.04	27.38	<0.001	0.79
	B2	1.01	0.04	28.58	<0.001	0.79
	B1	1.02	0.04	27.63	<0.001	0.79
Professional growth	D5	1.00				0.81
	D4	0.89	0.03	26.40	<0.001	0.77
	D3	0.94	0.04	25.63	<0.001	0.77
	D2	0.00	0.04	−0.03	0.973	−0.01
	D1	0.94	0.03	27.41	<0.001	0.78

### Reliability and validity of the modified scale with 15 items

After eliminating item D2, the reliability and validity of the modified scale were recalculated following the same procedures used for the 16-item scale.

#### Reliability

Alphas for the professional growth and the overall scale changed slightly, which were 0.85 and 0.89, respectively, while the other ones remained the same, indicating acceptable reliability. Please refer to Table 5 for the detailed information concerning the reliability for each domain and the entire scale.

**Table 5:** Factor loadings of the three-domain scale.

Factor	Item	Estimate	S.E.	C.R.	*p*	Standardize estimate
Value and expectation	A6	1.00				0.76
	A5	0.95	0.04	25.88	<0.001	0.73
	A4	0.94	0.04	24.82	<0.001	0.74
	A3	0.93	0.04	25.65	<0.001	0.73
	A2	0.94	0.04	23.86	<0.001	0.75
	A1	0.90	0.04	25.86	<0.001	0.73
Perceived confidence of teaching PE	B5	1.00				0.77
	B4	0.92	0.04	25.60	<0.001	0.75
	B3	0.97	0.04	27.06	<0.001	0.79
	B2	1.01	0.04	28.11	<0.001	0.79
	B1	1.03	0.04	27.38	<0.001	0.79
Professional growth	D5	1.00				0.81
	D4	0.90	0.04	25.85	<0.001	0.77
	D3	0.94	0.04	25.20	<0.001	0.77
	D1	0.93	0.04	26.67	<0.001	0.77

#### Construct validity

The modified scale CFA fit indices were all within acceptable range (CFI = 0.967, TLI = 0.961, GFI = 0.984, and RMSEA = 0.022), indicating acceptable construct validity for the hypothesized model. All factor loadings were greater than 0.7, the CR of the three factors was greater than 0.7, (value and expectation = 0.876, Perceived confidence of teaching PE = 0.875, and professional growth = 0.854).

#### Convergent and discriminant validity

**Figure 2:**
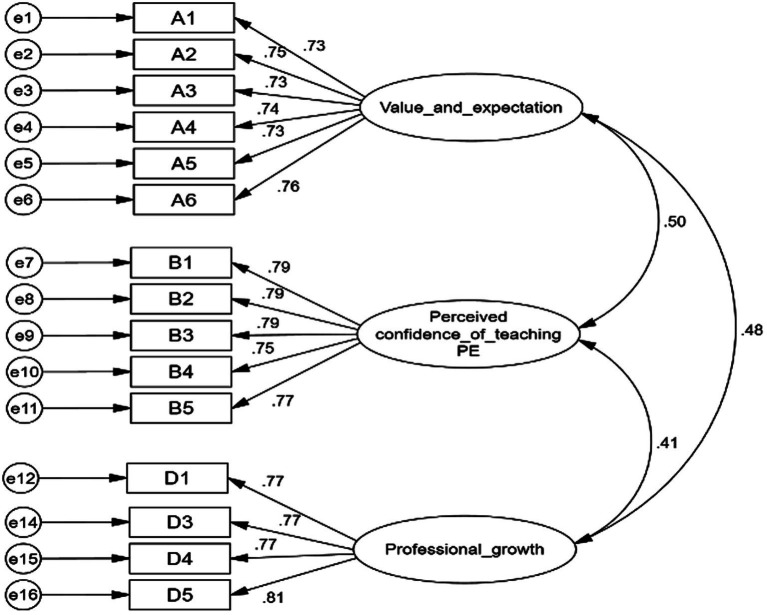
Modified structure model of PPET-TI scale.

The AVE was greater than 0.5 (AVE_ value and expectation = 0.541, AVE perceived confidence of teaching PE = 0.584, and AVE_ professional growth = 0.593), indicating that the questionnaire had good convergent validity. In addition, the square root of AVE is greater than any two-factor correlation coefficient, suggesting that the questionnaire has acceptable discriminant validity. The modified structure model of PPET-TI scale is illustrated in Figure 2.

## Discussion

Reliable and valid data collection instruments are very important to high quality research (Liu and Keating, 2022). With using valid and reliable data collection instruments, this strand of research could be greatly facilitated. This study aimed to revalidate the PPET-TI-Chinese scale in student teachers in PETE in order to provide more evidence of reliability and validity of the scale. To the best of our knowledge, the current study marks the first attempt to validate a TI scale in student teachers in PETE, which is a very special group, and usually is difficult to recruit due to their busy schedule. As might be expected, the hypothesis that the scale developed by Zhang (2017) may not fit the population of PETE student teachers was supported by our data given that the model fit indices were not within the acceptable range.

### Main finding of the investigation

Although the original scale developed by Zhang (2017) showed acceptable reliability and validity among preservice PE students, the revalidation of the scale using PETE student teachers suggested the need for modifications. The content validity study utilizing experts from both China and the US resulted in the deletion of the affect domain due to the low agreement among the experts. Because both Chinese and American experts participated in the content study, it is likely that the scale may be a better tool for its international use, marking the first attempt to develop a global scale for measuring TI in PE. Moreover, the deletion of the affect domain shortened the time needed to complete the questionnaire. Such an improvement may significantly enhance its utility given that many participants have a hectic schedule. Future research is needed to further examine its reliability and validity using other samples. Moreover, by deleting the unfitted item (i.e., D2 in Table 1), the model fit is improved. At the same time, the deletion of D2 does not affect the original structure, and still keeps the necessary components, which is essential for the scale. In general, in order to be able to cover the scope of the defined structure, a single remaining subdomain should consist of more than three items, which is the minimum number of items required for evaluation (Meyers et al., 2017). The revalidation of PPET-TI-Chinese in PETE student teachers showed acceptable reliability and validity after deleting the unfitted item.

### Main educational implications

As noted earlier, student teaching is the most crucial stage of PPETs’ professional learning (Browne, 2015). Their TI is of concern because it has the potential to influence PPETs’ career choices as well as the quality of their teaching as in-service teachers. As widely known, it is difficult to recruit student teachers because of the busy schedule of their student teaching, job searching, thesis writing, and travel to multiple schools for data gathering (Keating et al., 2021). The revalidation of PPET-TI-Chinese in PE student teachers provides us with a more effective tool to quantitatively examine TI.

#### Sample size and representativeness for scale development

The determination of sample size has always been a concern (Meyers et al., 2017), because no samples can completely represent their population. Especially in psychometric studies, scales are subject to individual subjective influence (Silverman, 2017). When the random factors remain unchanged, however, the conclusions of large samples are usually more accurate, and the error probability is generally lower than that of small samples. Therefore, although large samples require more time and energy, the conclusions obtained from larger samples are usually more representative of the targeted population. As well documented, it is not cost-efficient to use a larger sample size than necessary. To date, researchers have different suggestions on the proportion of participants to projects in the development of attitude scale. Ho (2006) believed that the conclusion drawn by the ratio of 10:1 was more acceptable. Others (Bentler and Wu, 2002) suggested a ratio of 5:1. In this study, the ratio of participants to scale items reached 63:1, which is much higher than the generally accepted ratio. Therefore, such a sample size is considered to be large. It is important to note that sample representativeness can have an impact on scale validity (Meyers et al., 2017). A psychometric scale, according to Ntoumanis and Vazou (2005), is a sample specific. As a result, the current study’s sample may still not represent the entire population of student teachers in PE. Therefore, future studies with different samples will be needed to revalidate the scale.

#### Strengths of the revalidated scale

First of all, there are fewer items (15 vs. 21), requiring less time to complete the questionnaire. Secondly, the revalidated scale is closer to the one developed by Liu and Keating (2022). Therefore, similar comparisons become possible. Finally, the revalidate scale is more general without specific characteristics associated with certain countries’ uniqueness. As such, PPET-TI-Chinese can be used more widely across nations.

### Future line of research

The revalidation of PPET-TI-Chinese in student teachers showed acceptable reliability and validity after deleting the unfitted item. In the future, the researchers may use the scale to track changes of PETI-TI and it can be used to investigate the effect of experimental research on the quality of PETE courses or student teaching. The teacher educators may also use the scale to examine PPETs’ current TI and provide interventions concerning improving PPETs’ TI. Schools may use the scale for PE teacher recruitment to identify those candidates with more positive TI.

The results of the study shed new light on the reliability and validity of the scale among student teachers in PE. The availability of a valid measure of PPET-TI-Chinese would permit the scale to be used in the assessment of the quality PPET preparation. Since no sample can represent the whole population, more representative samples need to be extracted in the future. The scale could also be translated into other languages to broad research on the topic in the future.

### Limitations

There are several limitations that should be noted. First of all, the study population in the original scale and the revalidated scale is PPETs in China. Therefore, further revalidation is needed if the scale is to be used in other populations. Second, no reverse-worded item was retained in the final version of the scale, even though reverse-worded items may help reduce response bias (Paulhus, 1991; Tenko and George, 2010). It is unfortunate that all the negatively worded items were identified as unfitted items. In addition, data concerning some main socio-demographic characteristics of the sample such as class size, type of internship school, and ethnicity were not included in our study. Cautions need to be exercised when generalizing and interpreting the results of the current study.

## Data availability statement

The raw data supporting the conclusions of this article will be made available by the authors, without undue reservation.

## Ethics statement

Ethical review and approval was not required for the study on human participants in accordance with the local legislation and institutional requirements. Written informed consent for participation was not required for this study in accordance with the national legislation and the institutional requirements.

## Author contributions

AY took the main responsibility for the research design, data collection, and writing the manuscript. YW and LY mainly contributed to the manuscript preparation, participated in the literature search, revision in terms of results, discussion, and provided their expertise in the data analyses. XH was responsible for coordinating the project, participated in data analyses, result interpretation, and writing the discussion. All authors contributed to the article and approved the final version.

## Conflict of interest

The authors declare that the research was conducted in the absence of any commercial or financial relationships that could be construed as a potential conflict of interest.

## Publisher’s note

All claims expressed in this article are solely those of the authors and do not necessarily represent those of their affiliated organizations, or those of the publisher, the editors and the reviewers. Any product that may be evaluated in this article, or claim that may be made by its manufacturer, is not guaranteed or endorsed by the publisher.

## References

[ref1] Adams K. Hean S. Sturgis P. Clark J. M. (2006). Investigating the factors influencing professional identity of first-year health and social care students. Learn. Health Soc. Care 5, 55–68. doi: 10.1111/j.1473-6861.2006.00119.x

[ref2] Anna L. Akhter I. Wang A. (2021). Why is There a Long-term Shortage of Music, Sports and Art Teachers? An Analysis of the Professional Role Development of Rural Primary School Teachers in China doi: 10.2139/ssrn.3844938.

[ref001] Azim M. (2017). Being a teacher: developing self-efficacy and teacher identity through self-reflective techniques in teacher education programs. J. Edu. Pract. 8, 7–14.

[ref3] Barkhuizen G. (2017). Reflections on Language Teacher Identity Research. New York: Routledge.

[ref4] Beauchamp C. Thomas L. (2009). Understanding teacher identity: an overview of issues in the literature and implications for teacher education. Camb. J. Educ. 39, 175–189. doi: 10.1080/03057640902902252

[ref5] Beijaard D. Meijer P. C. Verloop N. (2004). Reconsidering research on teachers’ professional identity. Teach. Teach. Educ. 20, 107–128. doi: 10.1016/j.tate.2003.07.001

[ref6] Bentler P. M. Wu E. J. C. (2002). EQS 6 for Windows User’s Guide. Encino, CA: Multivariate Software.

[ref7] Berry B. Shields P. M. (2017). Solving the teacher shortage: revisiting the lessons we’ve learned. Phi Delta Kappan 98, 8–18. doi: 10.1177/0031721717708289

[ref8] Browne T. (2015). A case study of student teachers’ learning and perceptions when using tablet applications teaching physical education. Asia-Pacific J. Health Sport Phys. Educ. 6, 3–22. doi: 10.1080/18377122.2014.997858

[ref9] Browne M. V. Cudeck R. (1992). Alternative ways of assessing model fit. Sociol. Methods Res. 21, 230–258. doi: 10.1177/0049124192021002005

[ref10] Byrne B. M. (2009). Structural Equation Modeling with AMOS: Basic Concepts, Applications, and Programming, 2nd Edn. New York: Mahwah, NJ: Lawrence Erlbaum Associates.

[ref11] Chen D. (2020). Comparative study on preservice training of physical education teachers in China and United States. J. Nanjing Sports Instit. 19, 41–47.

[ref12] Chong S. Ling L. E. Chuan G. K. (2011). Developing student teachers’ professional identities--an exploratory study. Int. Educ. Stud. 4, 30–38. doi: 10.5539/ies.v4n1p30

[ref13] Curran P. G. (2016). Methods for the detection of carelessly invalid responses in survey data. J. Exp. Soc. Psychol. 66, 4–19. doi: 10.1016/j.jesp.2015.07.006

[ref14] da Cunha M. A. Batista P. Graça A. (2014). Pre-service physical education teachers’ discourses on learning how to become a teacher: [re]constructing a professional identity based on visual evidence. Open Sports Sci. J. 7, 141–171. doi: 10.2174/1875399X01407010141

[ref15] Day C. Gu Q. (2007). Variations in the conditions for teachers’ professional learning and development: sustaining commitment and effectiveness over a career. Oxf. Rev. Educ. 33, 423–443. doi: 10.1080/03054980701450746

[ref16] Dowling F. (2011). ‘Are PE teacher identities fit for postmodern schools or are they clinging to modernist notions of professionalism?’ A case study of Norwegian PE teacher students’ emerging professional identities. Sport Educ. Soc. 16, 201–222. doi: 10.1080/13573322.2011.540425

[ref17] Du T. D. (2019). Service-learning within field experience of physical education teacher education in South Africa: experiences of pre-service and in-service teachers. South African J. Res. Sport Physic. Educ. Recreat. 41, 13–29. doi: 10.10520/EJC-14fdd49772

[ref18] Ferry M. (2018). Physical education preservice teachers’ perceptions of the subject and profession: development during 2005–2016. Phys. Educ. Sport Pedagog. 23, 358–370. doi: 10.1080/17408989.2018.1441392

[ref19] Heffernan K. A. Newton K. J. (2019). Exploring mathematics identity: an intervention of early childhood preservice teachers. J. Early Childhood Teacher. Educ. 40, 296–324. doi: 10.1080/10901027.2019.1590484

[ref20] Ho R. (2006). Handbook of Univariate and Multivariate Data Analysis and Interpretation. New York: Taylor & Francis

[ref21] Hong J. Y. (2010). Pre-service and beginning teachers’ professional identity and its relation to dropping out of the profession. Teach. Teach. Educ. 26, 1530–1543. doi: 10.1016/j.tate.2010.06.003

[ref22] Kaplan A. Garner J. K. (2017). A complex dynamic systems perspective on identity and its development: the dynamic systems model of role identity. Dev. Psychol. 53, 2036–2051. doi: 10.1037/dev0000339, PMID: 29094968

[ref23] Kaplan A. Garner J. Semo S. (2015). Teacher role-identity and motivation as a dynamic system. Paper presented at the annual meeting of the 2015 American Educational Research Association, Chicago, IL.

[ref24] Keating X. D. Liu J. Liu X. Colburn J. Guan J. Zhou K. (2021). An analysis of Chinese preservice physical education teachers’ beliefs about the physical education profession. J. Teach. Phys. Educ. 40, 58–65. doi: 10.1123/jtpe.2019-0095

[ref25] Keating X. D. Zhou K. Liu J. Shangguan R. Fan Y. Harrison L. (2017). Research on preservice physical education teachers’ and preservice elementary teachers’ physical education identities: a systematic review. J. Teach. Phys. Educ. 36, 162–172. doi: 10.1123/jtpe.2016-0128

[ref26] Keith T. Z. Reynolds M. R. (2018). “Using confirmatory factor analysis to aid in understanding the constructs measured by intelligence tests” in Contemporary Intellectual Assessment: Theories, Tests, and Issues, 4th Edn. (New York, NY: The Guilford Press), 853–900.

[ref27] Kelchtermans G. Vandenberghe R. (1994). Teachers’ professional development: a biographical perspective. J. Curric. Stud. 26, 45–62. doi: 10.1080/0022027940260103

[ref28] Klein D. F. (2004). Beyond significance testing: reforming data analysis methods in behavioral research. Can. Psychol. 45, 317–319. doi: 10.1037/h0087004

[ref29] Lee O. Jo K. (2016). Preservice classroom teachers’ identity development in learning to teach physical education. Asia Pac. Educ. Res. 25, 627–635. doi: 10.1007/s40299-016-0290-5

[ref30] Liu J. (2019). Understanding preservice physical education teachers’ teacher identity [Doctoral dissertation].

[ref31] Liu J. Keating X. D. (2022). Development of the pre-service physical education teachers’ teacher identity scale. Eur. Phys. Educ. Rev. 28, 186–204. doi: 10.1177/1356336X211028832

[ref32] Margarida A. Ana P. Amandio G. Paula B. (2012). Practicum as a space and time of transformation: self-narrative of a physical education pre-service teacher. US-China Educ. Rev. B 7, 665–674.

[ref33] McDonald R. P. (1999). Test Theory: A Unified Treatment. New York: Lawrence Erlbaum.

[ref34] Melnychuk N. Robinson D. B. Lu C. Chorney D. Randall L. (2011). Physical education teacher education (PETE) in Canada. Can. J. Educ. 34, 148–168.

[ref35] Meyer J. P. Allen N. J. Smith C. A. (1993). Commitment to organizations and occupations: extension and test of a three-component conceptualization. J. Appl. Psychol. 78, 538–551. doi: 10.1037/0021-9010.78.4.538

[ref37] Meyers L. S. Gamst G. C. Guarino A. J. (2017). Applied Multivariate Research: Design and Interpretation, 3rd Edn. Thousand Oak, CA: Sage.

[ref38] Ntoumanis N. Vazou S. (2005). Peer motivational climate in youth sport: measurement development and validation. J. Sport Exerc. Psychol. 27, 432–455. doi: 10.1123/jsep.27.4.432

[ref39] Paulhus D. (1991). “Measurement and control of response bias” in Measures of Personality and Social Psychological Attitudes. eds. Robinson J. P. Shaver P. R. Wrightsman L. S. (Cambridge, MA: Academic Press), 17–59.

[ref40] Preston C. C. Colman A. M. (2000). Optimal number of response categories in rating scales: reliability, validity, discriminating power, and respondent preferences. Acta Psychol. 104, 1–15. doi: 10.1016/S0001-6918(99)00050-5, PMID: 10769936

[ref41] Puchegger R. Bruce T. (2021). Reconceptualizing teacher identity: teachers’ becoming in the dynamic complexity of teaching situations. J. Teach. Phys. Educ. 40, 178–189. doi: 10.1123/jtpe.2019-0100

[ref42] Revilla M. A. Saris W. E. Krosnick J. A. (2014). Choosing the number of categories in agree–disagree scales. Sociol. Methods Res. 43, 73–97. doi: 10.1177/0049124113509605

[ref43] Rossi T. Lisahunter C. E. Macdonald D. (2016). Workplace Learning in Physical Education: Emerging Teachers’ Stories from the Staffroom and Beyond. London: Routledge.

[ref44] Samuel M. Stephens D. (2000). Critical dialogues with self: developing teacher identities and roles — a case study of south African student teachers. Int. J. Educ. Res. 33, 475–491. doi: 10.1016/S0883-0355(00)00030-6

[ref45] Silverman S. (2017). Attitude research in physical education: a review. J. Teach. Phys. Educ. 36, 303–312. doi: 10.1123/jtpe.2017-0085

[ref46] Sirna K. Tinning R. Rossi T. (2010). Social processes of health and physical education teachers’ identity formation: reproducing and changing culture. Br. J. Sociol. Educ. 31, 71–84. doi: 10.1080/01425690903385501

[ref47] Solmon M. A. Worthy T. Lee A. M. Carter J. A. (1991). Teacher role identity of student teachers in physical education: an interactive analysis. J. Teach. Phys. Educ. 10, 188–209. doi: 10.1123/jtpe.10.2.188

[ref48] Song G. Wei S. (2006). A study on influencing factors on teacher’s professional identity. Psychol. Dev. Educ. 1, 80–86.

[ref49] Tenko R. George A. M. (2010). Introduction to Psychometric Theory. New York: Routledge

[ref50] US Department of Education (2019). 2020 Title II reports national teacher preparation data. Available at: https://title2.ed.gov/Public/Home.aspx

[ref51] Vähäsantanen K. (2015). Professional agency in the stream of change: understanding educational change and teachers’ professional identities. Teach. Teach. Educ. 47, 1–12. doi: 10.1016/j.tate.2014.11.006

[ref52] Vámos Á. Doczi T. (2015). Everyday physical education: functional and dysfunctional consequences in Hungarian public education. Phys. Cult. Sport. Stud. Res. 67, 20–30. doi: 10.1515/pcssr-2015-0020

[ref53] Virta J. Hökkä P. Eteläpelto A. Rasku-Puttonen H. (2019). Professional identity among student teachers of physical education: the role of physicality. Eur. J. Teach. Educ. 42, 192–210. doi: 10.1080/02619768.2019.1576628

[ref002] Wagoner C. L. (2015). Measuring music teacher identity: self-efficacy and commitment among music teachers. Bull. Counc. Res. Music Educ. 205, 27–49. doi: 10.5406/bulcouresmusedu.205.0027

[ref54] Wang X. Liu H. (2017). Comparative study on training mode of preservice physical education teachers between China and US. J. Nanjing Sports Instit. 16, 16–19. doi: 10.3969/j.issn.1672-1365.2008.05.045

[ref55] Wang D. Zhang L. J. (2021). Sustainability as a goal in teaching workforce retention: exploring the role of teacher identity construction in preservice teachers’ job motivation. Sustainability 13:2698. doi: 10.3390/su13052698

[ref56] Ward P. (2019). The teacher pipeline for PETE: context, pressure points, and responses. J. Teach. Phys. Educ. 38, 4–13. doi: 10.1123/jtpe.2018-0008

[ref57] West S. G. Finch J. F. Curran P. J. (1995). “Structural equation models with nonnormal variables: problems and remedies,” in Structural Equation Modeling: Concepts, Issues, and Applications. (Thousand Oaks, CA: Sage Publications, Inc.), 56–75.

[ref58] Woods A. M. Ayers S. F. (2019). PETE recruitment and retention: current state of affairs. J. Teach. Phys. Educ. 38, 1–3. doi: 10.1123/jtpe.2018-0208

[ref59] Wrench A. Garrett R. (2012). Identity work: stories told in learning to teach physical education. Sport Educ. Soc. 17, 1–19. doi: 10.1080/13573322.2011.607909

[ref60] Zhang R. (2017). A Scale Study on Teacher Identity for Pre-service Physical Education Teacher. master’s thesis. Zhengzhou: Henan University.

[ref61] Zhang W. Meng H. Yang S. Liu D. (2018). The influence of professional identity, job satisfaction, and work engagement on turnover intention among township health inspectors in China. Int. J. Environ. Res. Public Health 15:988. doi: 10.3390/ijerph15050988, PMID: 29757985 PMC5982027

[ref003] Zhou K. Wang C. Zhou Y. (2012). Research on the teacher identity structure and scale formation of physical education teachers. J. Beijing Sport Univ. 35, 93–98. doi: 10.19582/j.cnki.11-3785/g8.2012.03.020

